# Echocardiography at a Tertiary Centre in Northern Ghana: A Retrospective Review

**DOI:** 10.7759/cureus.97212

**Published:** 2025-11-19

**Authors:** Dzifa Ahadzi, Kenneth M Dam, Abdul-Subulr Yakubu

**Affiliations:** 1 Internal Medicine, Tamale Teaching Hospital, Tamale, GHA; 2 Public Health, Tamale Teaching Hospital, Tamale, GHA

**Keywords:** cardiology, echocardiography, ghana, heart failure, hypertensive heart disease

## Abstract

Background

Transthoracic echocardiography (TTE) is a highly versatile and relatively affordable initial cardiac imaging modality that is useful for diagnosing cardiac structural and functional abnormalities. We present data of patients referred to a tertiary facility in northern Ghana for echocardiography. These findings provide insight into the spectrum of cardiovascular diseases prevalent in contemporary northern Ghana.

Methods

This was a cross-sectional retrospective review of data of patients presenting to the Cardiology Unit of the Tamale Teaching Hospital for TTE between October 2022 and September 2024.

Results

A total of 1,111 echocardiographic studies were performed, comprising 937 (84.3%) adult studies. Over half of adult patients referred for an echocardiogram were female (516 (55.1%)); however, male patients were significantly older (56±17 years versus 53±19 years, p=0.007). The commonest indication for adult echocardiograms was a clinical suspicion of heart failure (HF) (412 (44%)). Among adult patients referred with a clinical suspicion of HF, hypertensive heart disease (HHD) was the commonest etiology (109 (26.5%)). HHD was also the commonest primary echocardiographic diagnosis amongst the adult population (238 (25.4%)). Echocardiography confirmed the clinical suspicion of the referring clinician in 332 (35.4%) of adult patients.

Conclusion

The commonest indication for adult echocardiography was HF with HHD being the commonest underlying etiology. Our findings align with national trends of HHD being the most common cause of HF among Ghanaian adults. Notably, a significant proportion of echocardiograms were normal despite clinical suspicion of HF. A generally low diagnostic yield/confirmation rate underscores the need for training and local guidelines on appropriate use criteria for echocardiography. Further studies are needed to explore these findings on a national scale.

## Introduction

Transthoracic echocardiography (TTE) is the most requested cardiac imaging modality as it is highly versatile, relatively affordable, and carries minimal risk to the patient [1]. This cardiac imaging approach uses ultrasound technology and can be performed in various modes to acquire accurate data of cardiac structure and function for clinical decision-making [1]. Basic TTE using two-dimensional (2D) imaging and Doppler echocardiographic techniques is often sufficient for initial evaluation of patients referred for cardiac imaging [2]. A comprehensive TTE examination provides a detailed assessment of cardiac structure and function and is useful for the diagnosis of a broad range of cardiovascular diseases (CVDs) [1]. Beyond diagnostics, TTE is often needed to guide appropriate management, for follow-up, risk stratification, and prognostication [3]. Regional cardiac imaging data may also provide insights into the local burden of CVDs in the population.

The systematic review and meta-analysis on the prevalence of CVDs and risk factors in Ghana by Doku et al. showed a pooled prevalence of CVD of 10.34% with the commonest risk factors being hypertension, diabetes, unemployment, male gender, and older age [4]. Of 16 included studies, only one was conducted in Northern Ghana, where the prevalence of self-reported CVD, including heart failure (HF), myocardial infarction, and stroke, was 1.6% [5]. On a national scale, however, hypertensive heart disease (HHD) was the commonest CVD with a pooled prevalence of 25.1%, followed by stroke (24.3%) and valvular heart disease (19.7%) [4].

In northern Ghana, echocardiographic services have historically been limited due to a shortage of trained echocardiographers. Additionally, there is a paucity of data on CVDs in the region. To address this gap, the Cardiology Unit of Tamale Teaching Hospital began operations in June 2022, staffed by two certified cardiologists trained in comprehensive TTE. Since its inception, the unit has provided TTE services, receiving referrals from Tamale and other regions in northern Ghana. This study describes experience with echocardiography at Tamale Teaching Hospital and also characterises the spectrum of findings among adult patients referred for echocardiography.

## Materials and methods

Study setting

Tamale Teaching Hospital is a large tertiary referral center in Northern Ghana serving five major regions in the country, representing a population of over four million people. The hospital offers several specialist services including Cardiology Services under the Cardiology Unit of the Department of Internal Medicine and Therapeutics. The Cardiology Unit began operations in June 2022 with non-invasive cardiac diagnostic services including TTE, standard 12-lead electrocardiogram (ECG), Holter ECG monitoring, and ambulatory blood pressure monitoring. The unit is staffed by two Cardiologists, three dedicated nurses and five ECG technicians. TTE services are offered for inpatients as well as outpatients within the catchment area of Northern Ghana and beyond. On average, 80 TTE procedures are performed a month. 

Study design and population

This study was a cross-sectional retrospective review of archived echocardiographic data of patients presenting to the Cardiology Unit of the Tamale Teaching Hospital for TTE between October 2022 and September 2024. Most echocardiogram requests were received from the hospital's internal medical and pediatric departments, while others were from other departments in the hospital and other healthcare facilities across the region. Due to the retrospective nature of the study, informed consent was waived.

Inclusion and Exclusion Criteria

All patients presenting to the Cardiology Unit for a TTE over the study period were included. Patients with incomplete or inconclusive echocardiographic studies were excluded from the analysis.

Echocardiography Protocol

Prior to echocardiography, blood pressure (BP) was measured using a calibrated automatic sphygmomanometer (Omron M2, Omron Healthcare products, Matsusaka City, Japan). A mechanical round dial weighing scale (ADE M306800, Germany) and a mechanical height measure (ADE MZ10017, Germany) were used to measure weight and height, respectively. Echocardiographic studies were performed using a Sonoscape 22 Ultrasound system (Sonoscape Medical Corp., Shenzhen, China) equipped with a 3.5MHz cardiac transducer. Complete studies were performed by two certified cardiologists.

All study participants were evaluated using TTE in 2D mode, motion mode (M-mode) and Doppler analysis after pre-procedure counselling was done by the cardiologist. Standard echocardiographic evaluation was performed as per the recommendation of the American Society of Echocardiography (ASE)/European Association of Cardiovascular Imaging (EACVI). Normal limits for echocardiographic measurements were defined by the reference limits set by these societies [6,7]. Views interrogated include the parasternal and apical views in the lateral decubitus position and subsequently the subcostal and suprasternal views in the supine position. The inner edge-to-inner edge technique was used to measure linear dimensions of the left ventricular (LV) and right ventricular outflow tract in the parasternal long axis (PLAX) view in M-mode and/or 2D. The LV internal diameter in systole (LVIDs) and diastole (LVIDd), interventricular septum in diastole (IVSd) and LV posterior wall in diastole (LVPWd) were thus acquired in the appropriate frames. The Teichholz method was used to estimate left ventricular ejection fraction (LVEF) and LV volumes from linear measurements obtained in the PLAX view [2]. 

HHD was diagnosed in patients with a history of hypertension and structural heart changes including left ventricular hypertrophy and/or left atrial dilatation and LV diastolic and/or systolic dysfunction [8]. LV systolic dysfunction was diagnosed with an LVEF <50%, with ranges of 41 - 49% indicating mild LV systolic dysfunction [6,9]. LV diastolic dysfunction was diagnosed using limits set by Nagueh et al. [10].

Rheumatic heart disease (RHD) was diagnosed with the World Heart Federation RHD diagnostic criteria [11]. Dilated cardiomyopathy was diagnosed in patients with dilated LV and systolic dysfunction unexplained solely by abnormal loading conditions [12]. Ischaemic heart disease (IHD) was a probable diagnosis in patients with a history of ischemic chest pain, electrocardiographic changes and regional wall motion abnormalities [13]. Pericardial diseases constituted pericardial thickening, pericardial effusion (echo-free space between the two layers of pericardium) and constrictive pericarditis diagnosed using standard diagnostic echocardiographic criteria [14].

Right ventricular dysfunction was diagnosed when tricuspid annular plane systolic excursion at the tricuspid lateral annulus was less than 17mm [15]. Pulmonary hypertension was diagnosed when the right ventricular systolic pressure was greater than 35mmHg in the absence of pulmonary stenosis [15]. Peripartum cardiomyopathy was diagnosed in women presenting with HF secondary to an idiopathic cardiomyopathy with LV systolic dysfunction (LVEF <45%), towards the end of pregnancy or in the months following delivery [16]. Congenital heart diseases were diagnosed according to European Society of Cardiology recommended classifications [17].

The final echocardiographic diagnosis was based on major structural abnormalities detected in combination with clinical history and pertinent physical examination findings.

Data collection

Archived echocardiographic reports of patients referred to the Cardiology Unit of the Tamale Teaching Hospital were extracted by research assistants who were trained with explicit definitions for all variables. Demographic data (age and sex), anthropometric data for adults (blood pressure, weight, and height), and echocardiographic data (including indication for echocardiography, echocardiographic parameters of cardiac structure and function and final echocardiographic diagnosis) were collected. All entries were systematically reviewed by a cardiologist. All identified discrepancies were flagged and resolved through a consensus review by the principal investigators.

Statistical analysis

Data was collected in an Excel Spreadsheet and then imported into IBM SPSS Statistics for Windows, Version 20 (Released 2011; IBM Corp., Armonk, New York, United States) for analysis. Participants’ characteristics were summarised as mean and standard deviation (SD) for continuous variables and percentages for categorical variables. Chi-square, Fisher’s exact, and Student’s t-test were used as appropriate to make comparisons between data collected from male and female participants. We calculated the confirmation rate by dividing the number of confirmed diagnosis in an echocardiogram by the total number of requests for each cardiac diagnostic category for which TTE requests were made. A two-sided p-value of <0.05 was considered significant in all inferential analyses.

Ethical considerations

Ethical approval for this study was granted by the Tamale Teaching Hospital Ethics Committee (TTHERC/05/07/23/01). 

## Results

A total of 1,111 echocardiographic studies were performed over the study period comprising 937 (84.3%) adult and 174 (15.7%) pediatric studies. Over half of adult patients referred for an echocardiogram were female (55.1%). Among adults, male patients were significantly older (56±17 years) with the largest age category being between 41 and 60 years of age (339 (36.2%)). There were no sex differences in BPs of study participants. Among pediatric patients, the commonest indication for echocardiograms was a clinical suspicion of congenital heart disease, accounting for 132 (75.9%) requests, whilst HF was the commonest reason for an echocardiogram request in adults, 413 (44.1%).

Amongst adult patients, significantly more female patients than male patients were referred for an echocardiogram on account of a clinical suspicion of HF (244 (59.2%) vs 168 (40.8%), p=0.024), whereas more male patients were referred for evaluation of suspected IHD (36 (61%) vs 23 (39%), p=0.010) (Table 1).

**Table 1:** General characteristics of the adult study population DBP: Diastolic blood pressure; SBP: Systolic blood pressure; SD: Standard deviation †Missing data – 26 *Statistically significant p-value of <0.05 ‡Test statistic – Chi-square, Fisher’s Exact Test, Independent-Samples T-test used as appropriate

	Total (n=937)	Sex		
					Male	Female	p-value^‡^
		(n=421)	(n=516)				
		Age (years), Mean ± SD	54±19	56±17	53±19	0.007*
Age category (years), n (%)				
13-40	233 (24.9)	77 (33.0)	156 (67.0)	<0.001*
41-60	339(36.2)	167(49.3)	172(50.7)	0.045*
61-80	275(29.3)	144(52.4)	131(47.6)	0.003*
>80	90 (9.6)	33 (36.7)	57 (63.3)	0.097
Blood pressure^†^				
SBP category (mmHg), n (%)				
Non-elevated (<120)	412 (45.2)	186 (45.1)	226 (54.9)	0.574
Elevated (120-139)	300 (32.9)	126 (42.0)	174 (58.0)	0.365
Hypertension (≥140)	199 (22.0)	90 (45.2)	109 (54.8)	0.724
DBP category (mmHg), n (%)				
Non-elevated (<70)	264 (28.2)	115 (43.6)	149 (56.4)	0.826
Elevated DBP (70-89)	462 (49.3)	197 (42.6)	265 (57.4)	0.359
Hypertension (≥90)	185 (20.3)	90 (48.6)	95 (51.4)	0.165
SBP (mmHg), Mean ± SD	125±23	124±23	125±23	0.413
DBP (mmHg), Mean ± SD	78±15	78±15	78±15	0.954
Indication for echocardiography, n (%)				
Heart failure	412 (44.0)	168 (40.8)	244 (59.2)	0.024*
Hypertension	156 (16.0)	62 (39.7)	94 (60.3)	0.154
Suspected pulmonary embolism/right heart failure	73 (7.8)	37 (50.7)	36 (49.3)	0.303
Stroke	62 (6.6)	35 (56.4)	27 (43.6)	0.059
Suspected ischemic heart disease	59 (6.3)	36 (61.0)	23 (39.0)	0.010*
Suspected cardiomyopathy	36 (3.8)	12 (33.3)	24 (66.7)	0.154
Pre-operative assessment	33 (3.5)	18 (54.5)	15 (45.5)	0.258
Suspected valvular heart disease	23 (2.5)	10 (43.5)	13 (56.5)	0.887
Arrhythmia	22 (2.3)	11 (50.0)	11 (50.0)	0.629
None	14 (1.5)	7 (50.0)	7 (50.0)	0.701
Pericardial diseases	12 (1.3)	8 (66.7)	4 (33.3)	0.151
Congenital heart disease	6 (0.6)	2 (33.3)	4 (66.7)	0.696
Other	29(3.1)	15 (51.7)	14 (48.3)	0.455
Primary echocardiographic diagnosis, n (%)				
Normal	272(29.0)	114 (41.9)	158 (58.1)	0.001*
Hypertensive heart disease	238 (25.4)	113 (47.5)	125 (52.5)	0.360*
Cardiomyopathy	153 (16.3)	66 (43.1)	87 (56.9)	0.626
Valvular heart disease	71(7.6)	27 (38.0)	44 (62.0)	0.224
Right ventricular dysfunction ± pulmonary hypertension	68 (7.3)	22 (32.4)	46 (67.6)	0.030*
Ischemic heart disease	53 (5.7)	39 (73.6)	14 (26.4)	<0.001*
Pericardial diseases	27 (2.9)	15 (55.6)	12 (44.4)	0.260*
Aortic diseases	41 (4.4)	20 (48.8)	21 (51.2)	0.612
Congenital heart diseases	12 (1.3)	4 (33.3)	8 (66.7)	0.416
Other	2 (0.2)	1 (50.0)	1 (50.0)	0.885

**Figure 1:**
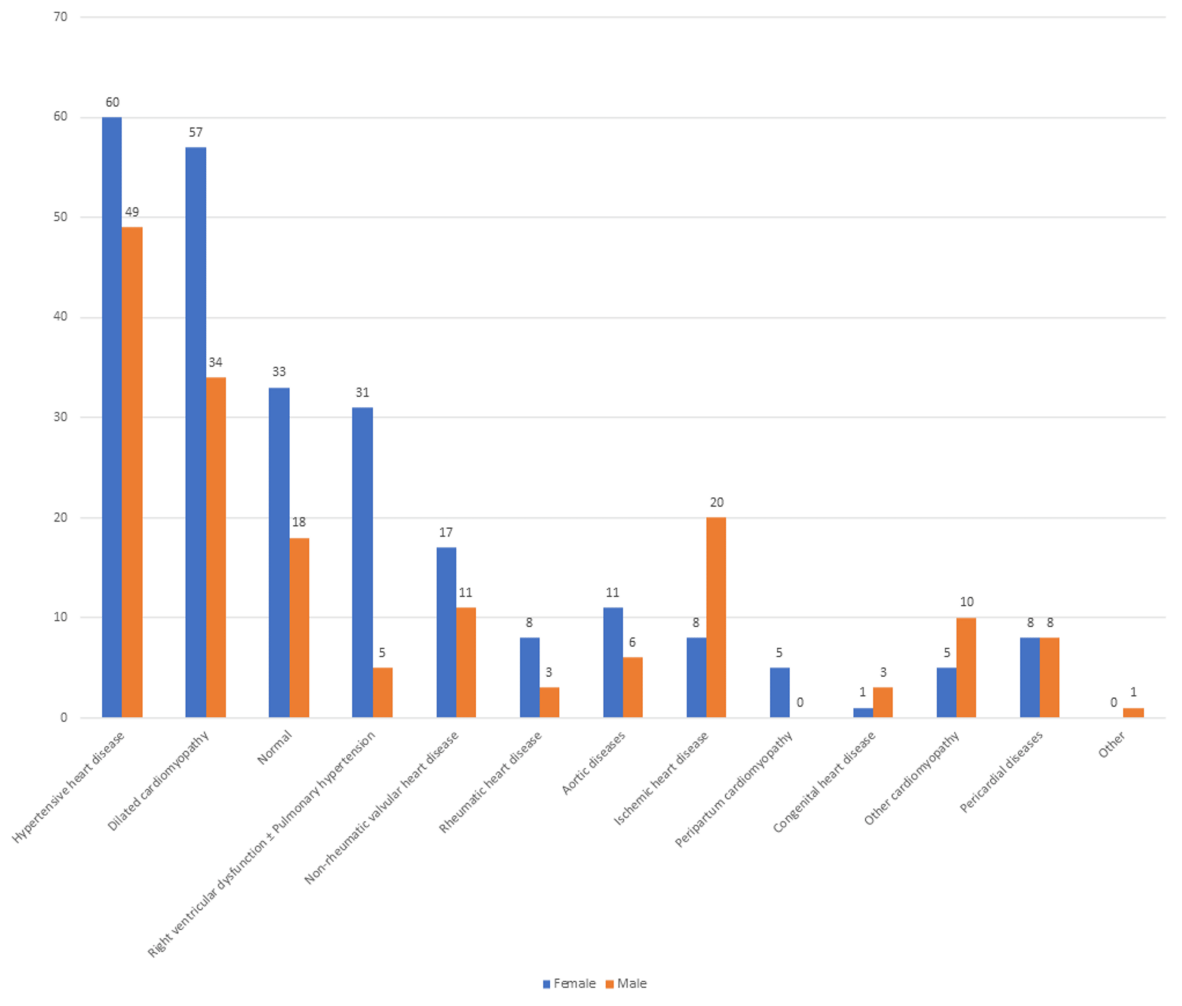
Echocardiographic findings among patients referred with a clinical suspicion of heart failure Number of patients referred with a clinical suspicion of heart failure (Total (N)= 412 - Male patients = 168, Female patients = 244)

The majority of echocardiograms performed were normal and the most common abnormality detected in both sex categories was HHD. HHD was the commonest underlying etiology of clinically suspected HF (Figure 1).

RHD comprised 18 out of 44 (41%) valvular heart diseases observed among female patients, and 4 out of 27 (14.8%) among male patients. Dilated cardiomyopathy constituted 49 out of 66 (74.2%) and 71 out of 87 (81.2%) of cardiomyopathies identified in male patients and female patients, respectively. Nine (13.6%) of 66 cardiomyopathies in female patients were consistent with peripartum cardiomyopathy.

A total of 222 study participants had LV systolic dysfunction (LVEF <50%), with 119 (53.6%) being female. LVEF was reduced (≤40%) in 64(53.9%) of female patients and mildly reduced (41-49%) in 55 (46.1%) of female patients, with no sex differences in prevalence of LV systolic dysfunction (p=0.860).

**Figure 2:**
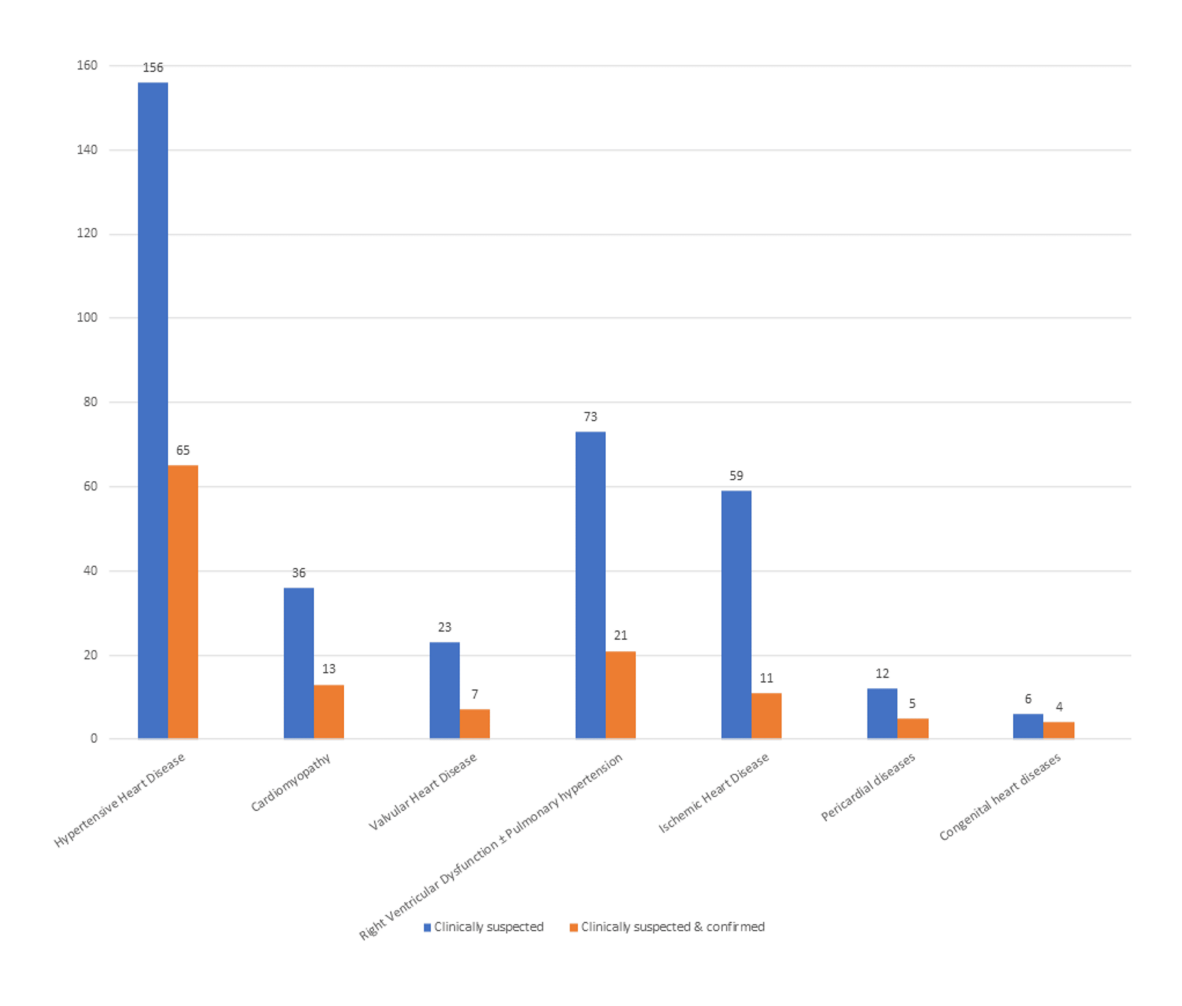
Diagnostic value of echocardiographic studies

Comparing indications for echocardiographic studies with primary echocardiographic diagnosis, echocardiography had the least diagnostic value for a clinical suspicion of IHD (confirmation rate was 18.6%) (Figure 2). The general confirmation rate of clinically suspected cardiac disease was 34.5% (126 out of 365).

## Discussion

The findings of our study highlight the value of echocardiography in the evaluation of patients with CVDs in a resource-limited setting. The most common indication for adult echocardiography in this study was a clinical suspicion of HF (44.1%), with this suspicion being significantly higher in female patients than in male patients. The commonest etiology of HF for both sexes was hypertension. Hypertension was the second-highest reason for a request for an echocardiogram in this population, with no sex differences observed. 

In a study by Wiafe et al. in Ghana, the commonest indication for echocardiography was “cardiac assessment”, whilst Oyedeji et al. reported hypertension as the commonest indication in a Nigerian population [3,13]. These findings from Oyedeji et al. align with continental CVD patterns, where hypertension predominates as both the most prevalent cardiovascular risk factor and the leading driver of cardiovascular morbidity [18]. Adbullahi et al. report HHD as the commonest finding amongst their cohort of patients referred to a tertiary centre in Nigeria for echocardiography, but do not link this echocardiographic diagnosis to HF [19].

The high volume of referrals for evaluation of suspected HF may reflect clinicians' increasing appreciation of the importance of echocardiography in the clinical evaluation of patients with symptoms and signs suggestive of HF. Though the prevalence of HF in the African region is not precisely known, recent epidemiological studies suggest that the disease burden is increasing in the region whilst stabilising or even declining in high-income countries [20]. Hypertension is the commonest underlying etiology of HF in Africa, and studies conducted in Ghana and Nigeria show a similar etiological trend [21-25]. In patients with a clinical suspicion of HF, echocardiography is useful in determining the presence of underlying structural and functional cardiac abnormalities [26]. This may aid the clinician in confirming the clinical suspicion, phenotyping the patient according to LVEF, determining if additional evaluation, such as natriuretic peptide testing, is needed, and planning tailored therapeutic interventions [26].

In this resource-limited setting with limited diagnostic capacity for obstructive coronary artery disease, a probable diagnosis of IHD on echocardiogram was based on a suggestive clinical context, including a history of ischemic chest pain, documented history of an acute coronary syndrome, presence of cardiovascular risk factors, and ischemic electrocardiography changes. Agyekum et al., Abdullahi et al., and Onwuchekwa and Asekomeh document a similar approach to diagnosing ischemic heart disease in Ghana and Nigeria, respectively, due to limited access to invasive angiography [19,21,25]. There is a scarcity of robust regional data on IHD in Africa, likely due to diagnostic challenges including limited access to cardiac biomarker testing, electrocardiography, invasive angiography, and non-invasive ischemia testing [27]. Despite this major diagnostic limitation, the burden of IHD in the African region is estimated to be on the rise based on global epidemiological data, including estimates from the Global Burden of Disease Study [27,28]. In this study, significantly more males than females had an echocardiographic diagnosis of probable IHD, consistent with regional data showing a higher disease burden in males [29,30]. Abdulllahi et al. however report a contrasting finding of a significantly higher burden of IHD in females [19]. In this study, approximately one out of every five patients presenting for echocardiography on account of a clinical suspicion of IHD had the diagnosis corroborated on echocardiogram. Oyedeji et al. similarly report a lower clinical suspicion of IHD compared with echocardiographic diagnosis (1.2% vs 2.4%) [3]. Wiafe et al., in Ghana, report a clinical suspicion of IHD of 1.01% amongst referred patients but do not state the confirmation rate [13]. The observed discrepancies between clinically suspected IHD and echocardiographic diagnosis may be due to limited capacity to diagnose acute coronary syndromes or evaluate for chronic coronary syndromes in resource-limited settings, leading to heavy reliance on echocardiographic to confirm clinically suspected IHD. 

Though global epidemiologic data on HF show that IHD is the leading etiology, the prevalence rates are highest in high-income regions (40-60%) and lowest in Africa (12%) [31,32]. In this study, 6.8% of patients referred for echocardiography on account of HF had a probable diagnosis of IHD. Compared with other studies in Ghana, this finding was similar to data from Appiah et al. in 2017 (7.2%) [22], higher than that reported by Owusu et al. in 2013 (2.3%) [24], and lower than 13.6% documented in the study by Agyekum et al. in 2023 [21]. In Nigeria, Ogah et al. and Onwuchekwa and Asekomeh report even lower rates of IHD as the underlying etiology of HF in 0.2 and 0.4% of cases, respectively [25,33]. In these resource-limited settings, differing demographic profiles of study participants and the varying diagnostic capacities of each study centre may account for the differences observed.

Echocardiography provides the highest clinical value when it is requested in the appropriate clinical context [34]. Requesting clinicians must therefore provide adequate patient information to enable the echocardiographer to perform a thorough evaluation to support clinical decision-making [34]. In this cohort of patients, very few requests (1.5%) had no indication stated. This contrasts with studies by Oyedeji et al. and Wiafe et al. in Nigeria and Ghana, respectively, where all requests were labelled [3,13]. However, the designation of “cardiac assessment” as an equally vague indication was common in the study by Wiafe et al., highlighting the need for local education on appropriate use criteria for echocardiography and the value of a well-written request in providing clinical answers. Bethge et al., in a study in the United States, record the recognition of a cardiac abnormality in approximately 22% of patients who had an echocardiogram requested, with only 2.5% experiencing a change in management because of the abnormality detected [35]. Wiafe et al. report a confirmation rate of 70% in a specialist cardiac outpatient clinic in Ghana, which is higher than our confirmation rate of 34.5% [13]. These disparities may be due to variations in cadres of referring clinicians from region to region, comparing the location of both study centres, with southern Ghana harbouring higher numbers of more specialised clinicians, including cardiologists and specialist physicians. They also highlight the need for training and algorithms to guide referring clinicians on appropriate use criteria guidelines to leverage the clinical and diagnostic value of echocardiography. We also found that the diagnostic value of echocardiography exceeded the clinical suspicion for all the major primary diagnoses examined demonstrating the ability of echocardiography to unmask silent cardiovascular diseases. This may result in unnecessary downstream testing and treatment or may alternatively aid in timeous clinical intervention to avert adverse outcomes, depending on the clinical context. 

Strengths and limitations

Our study represents the first of its kind in Northern Ghana and therefore provides unique epidemiological insights that may be useful in local CVD care planning. The retrospective design of the study limited the quantum of data that could be collected. Given the spectrum of cardiac abnormalities that were detected, the findings of this study underscore the importance of echocardiography as an initial cardiac imaging tool in patients with suspected structural and functional cardiac disease. The clinical value of these findings would have been better appreciated if follow-up data on their impact on patient care were collected.

## Conclusions

This study presents preliminary insights into the spectrum of echocardiographic findings in northern Ghana, where CVD data are scarce. Among patients referred to a tertiary facility in northern Ghana for echocardiography, a clinical suspicion of HF was the commonest indication and the commonest etiology for clinically suspected HF was hypertension. The confirmation rate of suspected cardiac disease was low. There is a need for local guidance on appropriate use criteria for echocardiography coupled with training for referring clinicians to improve the diagnostic yield of echocardiography in the region.
